# Giant Cell Tumor of Tendon Sheath of Distal Phalanx of Thumb: A Case Report

**DOI:** 10.31729/jnma.8182

**Published:** 2023-06-30

**Authors:** Puja Chaurasia

**Affiliations:** 1 Department of Surgery, Narayani Central Hospital Birgunj Pvt. Ltd., Birgunj, Parsa, Nepal

**Keywords:** *case reports*, *giant cell tumors*, *tendons*, *thumb*

## Abstract

Giant cell tumor of tendon sheath is an uncommon benign soft tissue tumor. Histopathological examination plays a crucial role in the definitive diagnosis of giant cell tumor although pre-operative imaging supports its suspicion. We report a case of a giant cell tumor of the tendon sheath in a 26-year-old man as a painless, firm, localized, slow-growing benign soft tissue tumor of the thumb; managed by complete excision. The patient continues to do well at 7 months post-surgery with no complaints and no signs of recurrence. Giant cell tumor of the phalanges is a locally aggressive entity; therefore delayed or missed diagnosis of giant cell tumor especially of the thumb distal phalanx can be extremely debilitating. Hence, high degree of suspicion and early en bloc resection is the key to its management.

## INTRODUCTION

Giant cell tumor of tendon sheath (GCTTS) is a benign, slow-growing, locally aggressive soft tissue tumor of the hand region with an overall incidence of 1 in 50,000 individuals.^1,2^ Etiology of the entity includes trauma, inflammation, metabolic disease, and neoplasia.^3,4^ It occurs at any age with female predominance in the third to fifth decade of life.^5^ Histopathological examination of the tumor with clinical examination and various radiological armamentariums aid in the diagnosis.^6^

## CASE REPORT

A 26-year-old male presented with complaints of swelling over the front of his right thumb. The lump was spontaneous, painless, and gradually progressive in size since he first noticed it 3 months back. He denied any history of trauma or prior occurrence of the lesion, fever, bruising, numbness, tingling, and purulent discharge. Clinical examination revealed a 3x2 cm firm, immobile, solitary mass attached to a tendon on the volar bulb of the right thumb of the dominant hand. The lump didn't extend into the subungual region and adjacent skin was normal. There were no signs of inflammation or discharge. The full range of motion of the distal interphalangeal joint could be obtained. There were no signs of neurosensory indifference. We thereafter made a differential diagnosis of either a giant cell tumor or a ganglionic cyst.

**Figure 1:**
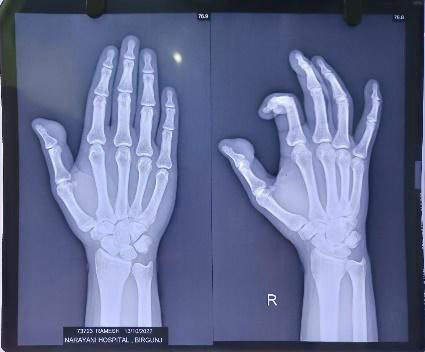
Posteroanterior X-ray of the right hand.

For investigation, a plain radiograph was obtained which showed a soft tissue swelling with normal bones; no fracture, dislocation, or destructive lesion (Figure 1).

An ultrasound examination revealed a hypoechoic complex solid-looking cystic lesion measuring about 21x16 mm at the ventral aspect of the right thumb with floating low-level echoes. No intralesional vascularity was observed. Adjacent vessels were normal.

**Figure 2:**
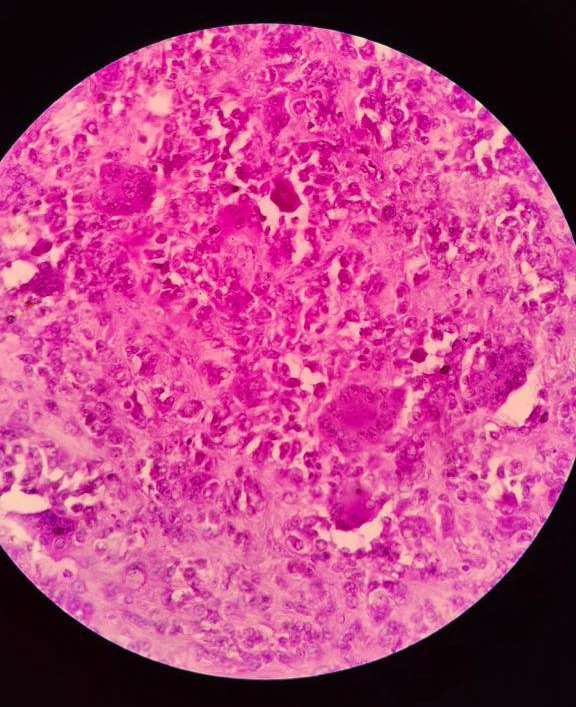
Hematoxylin and eosin staining of the resected specimen showed numerous multinucleated giant cells and mononuclear cells at 20x magnification.

The histopathological examination demonstrated features of giant cell tumor tendon sheath. Cut section examination revealed a greyish-white mass with cystic spaces. It showed tissue exhibiting the proliferation of cells. The cells were having round to oval and spindle nuclei. The cells were arranged in fascicles and storiform pattern focally. Numerous multinucleated giant cells were seen dispersed in the stroma. Areas of edema were also seen (Figure 2).

**Figure 3:**
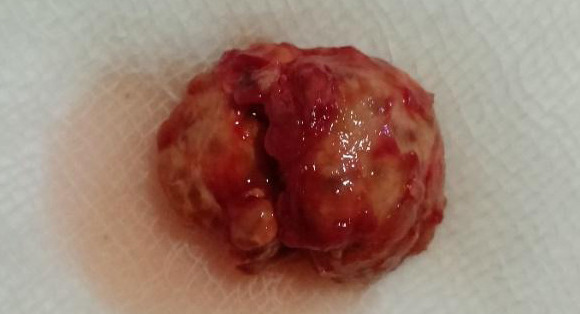
Tumor after complete resection.

Intra-operatively, a volar oblique incision was made on the right thumb under local anesthesia. The dissection was then carried into the subcutaneous tissue. A large mass measuring 3x2x0.5 cm grey-brown in color was completely removed (Figure 3).

The patient was discharged on the same day with a course of antibiotics and analgesics.

**Figure 4:**
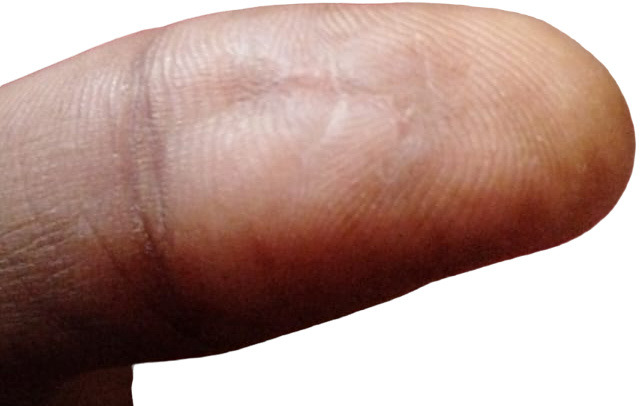
Post-operative image of the thumb at 7 months of GCTTS excision.

The patient was then evaluated 10 days later in the outpatient department. The patient had an uneventful post-operative course and he was advised for further follow-up at 6 weeks and 6 months. However, he continues to do well at 7 months post-surgery with no complaints and no signs of recurrence (Figure 4).

## DISCUSSION

This case was intended to report an uncommon case of a giant cell tumor tendon sheath of the distal phalanx of the thumb. Giant cell tumors most commonly affect the first three fingers, with the index finger being the most frequently involved, whereas, in this patient, the thumb was the site of the lesion which is uncommon.^6^'^8^ These tumors are most commonly found in the region of the distal interphalangeal (DIP) joint, followed by the proximal phalanx, and usually in the dominant hand similar to our case.^9^

GCTTS manifests clinically as a firm, painless, gradually growing focal mass likely to impair the function of the involved tendon and joint.^4,5^ A plain radiograph commonly detects any soft tissue swelling and also determines whether a fracture, dislocation, or destructive lesion exists.^5,6^ In this case, there was no joint restriction, fracture, dislocation, or bony destruction which may be due to early presentation to the hospital. Sonography can be used to distinguish between solid and cystic lesions, and their relationship with the surrounding structures and to detect satellite lesions.^6^ MRI is the preferred imaging modality, particularly for evaluating extra-articular manifestations.^5,10^ In this specific case, a hypoechoic complex solid-looking cystic lesion was detected on ultrasonography, however, MRI was not performed due to the unaffordability of the patient.

In our case, GCTTS and ganglion cyst were the differential diagnoses we came up with based on the history, clinical and radiological examination. An ultrasound revealed that a ganglion cyst was more likely than a giant cell tumor. Complete surgical excision is the primary treatment for GCTTS.^7,10^ In this patient, the mass was excised in toto. Because of the presence of a pseudo capsule, the tumor is frequently removed as a whole.^5^ However, after surgical excision, the tumor's characteristics were more suggestive of a giant cell tumor, indicating histopathological confirmation. A histopathological examination of the excised tissue was performed, which confirmed the diagnosis of GCTTS. Hence, histopathological examination is a must to confirm the diagnosis.^7,10^

Recurrence is a major risk factor. Given the reported high recurrence rate of up to 45%, long-term follow-up after excision is advised.^7,10^ The recurrence was significantly higher at the thumb interphalangeal (IP) and distal digital interphalangeal (DIP) joints.^1^ The reason may be the difficult excision of the tumor distally at the proximal interphalangeal (PIP) joint or the thumb IP and DIP joint due to the proximity of neurovascular structures to tumor margins.

The patient was then evaluated 10 days later in the outpatient department. The patient had an uneventful post-operative course and continues to do well at 7 months post-surgery with no complaints and no signs of recurrence which was in line with a previous study.^10^

This report presents a case of giant cell tumor tendon sheath of distal phalanx thumb and its diagnosis and treatment in a resource constraint country. The report also emphasizes the significance of histopathological examination for the confirmation of diagnosis.
